# Prevalence and Prediction of Obstructive Coronary Artery Disease in Patients Undergoing Primary Heart Valve Surgery

**DOI:** 10.5935/abc.20170135

**Published:** 2017-10

**Authors:** José Guilherme Cazelli, Gabriel Cordeiro Camargo, Dany David Kruczan, Clara Weksler, Alexandre Rouge Felipe, Ilan Gottlieb

**Affiliations:** 1 Instituto Nacional de Cardiologia (INC – MS), Rio de Janeiro, RJ – Brazil; 2 Instituto Estadual de Cardiologia Aloysio de Castro (IECAC), Rio de Janeiro, RJ – Brazil

**Keywords:** Coronary Artery Disease, Heart Valve Disease, Coronary Angiography, Computed Tomography Angiography

## Abstract

**Background:**

The prevalence of coronary artery disease (CAD) in valvular patients is similar to that of the general population, with the usual association with traditional risk factors. Nevertheless, the search for obstructive CAD is more aggressive in the preoperative period of patients with valvular heart disease, resulting in the indication of invasive coronary angiography (ICA) to almost all adult patients, because it is believed that coronary artery bypass surgery should be associated with valve replacement.

**Objectives:**

To evaluate the prevalence of obstructive CAD and factors associated with it in adult candidates for primary heart valve surgery between 2001 and 2014 at the National Institute of Cardiology (INC) and, thus, derive and validate a predictive obstructive CAD score.

**Methods:**

Cross-sectional study evaluating 2898 patients with indication for heart surgery of any etiology. Of those, 712 patients, who had valvular heart disease and underwent ICA in the 12 months prior to surgery, were included. The P value < 0.05 was adopted as statistical significance.

**Results:**

The prevalence of obstructive CAD was 20%. A predictive model of obstructive CAD was created from multivariate logistic regression, using the variables age, chest pain, family history of CAD, systemic arterial hypertension, diabetes mellitus, dyslipidemia, smoking, and male gender. The model showed excellent correlation and calibration (R² = 0.98), as well as excellent accuracy (ROC of 0.848; 95%CI: 0.817-0.879) and validation (ROC of 0.877; 95%CI: 0.830 - 0.923) in different valve populations.

**Conclusions:**

Obstructive CAD can be estimated from clinical data of adult candidates for valve repair surgery, using a simple, accurate and validated score, easy to apply in clinical practice, which may contribute to changes in the preoperative strategy of acquired heart valve surgery in patients with a lower probability of obstructive disease.

## Introduction

Coronary artery disease (CAD) in patients with valvular heart disease has the usual association with traditional risk factors. Nevertheless, the search for obstructive CAD is more aggressive in the preoperative period of patients with valvular heart disease, resulting in the indication of invasive coronary angiography (ICA) to almost all patients older than 35 years, because it is believed that coronary artery bypass surgery should be associated with valve replacement in the presence of obstructive CAD.

Angina is the major symptom, even though it can have other causes in valvular heart disease,^1^ such as left ventricular hypertrophy or overload. Association of obstructive CAD with the impaired heart valve, mainly the aortic valve, is common; however, increasing age has been shown to accompany a higher prevalence of CAD, regardless of the valve.^2,3^ Older patients tend to have degenerative aortic valve disease more often, but CAD does not differ between patients with aortic or mitral valve impairment in the same age group.^4^

The epidemiology of valvular heart disease is heterogeneous and has changed over the past decades in different countries. Rheumatic heart disease was the major cause of valvular heart disease until the mid-20th century, after which, with the widespread use of antibiotics and better access to health care, a substantial reduction in the incidence of that inflammatory valvular heart disease occurred in developed countries.^5^ The current prevalence of rheumatic valvular disease is estimated to be 2.5% in the USA and Canada, and 22% in Europe.^6^ Concomitantly, with the increase in life expectancy, the prevalence of age-related heart diseases increased, the degenerative etiology being the most common cause of valvular heart disease in developed countries.^7^ In addition, the higher mean age and, consequently, the higher number of chronic diseases and associated atherosclerotic risk factors increase the prevalence of CAD, which, in North-American and Anglo-Saxon patients with valvular heart disease ranges from 20% to 40%.^8,9^

In developing countries, rheumatic heart disease is still the major cause of valvular heart disease.^10^ In Brazil, its prevalence reaches 60.3%, with a mean age of 37 years.^7^ It usually affects young individuals, who have less risk factors for atherosclerosis, and, thus, lower prevalence of obstructive CAD.^11,12^

The guidelines suggest that, because of the impact of non-treated CAD, its diagnosis is paramount.^1^ Preoperative ICA is indicated to almost all patients older than 35 years, and non-invasive functional tests are not recommended because of their limited specificity. In the ACC/AHA guideline, coronary computed tomography angiography (CCTA) is suggested for patients with a low or intermediate pretest probability of CAD (class of recommendation IIa, level of evidence C), because of its high negative predictive value to exclude obstructive CAD.^13^

Stratification of obstructive CAD based on current indications does not seem to be the best strategy in our population. The ICA is a high-cost invasive procedure with widely documented morbidity and mortality. The development of tools to estimate the pretest probability of obstructive CAD, as performed in the general population, is urgent, to better select patients who will benefit from different preoperative strategies, therefore preventing the indiscriminate indication of unnecessary and invasive procedures, mainly in groups with a lower clinical probability of obstructive CAD.

This study was aimed at developing a predictive score for obstructive CAD in adult candidates for primary heart valve surgery, and at validating that score in an independent cohort of patients from another tertiary reference institution.

## Methods

### Selection of patients

The population studied comprises adults with primary acquired valvular heart disease from a tertiary reference hospital, submitted to heart valve replacement or repair surgery between 2001 and 2014.

### Inclusion criteria

This study included patients older than 18 years with primary acquired valvular heart disease, submitted to heart valve surgery between 2001 and 2014, who underwent ICA within 12 months from surgery.

### Data collection

Data were obtained retrospectively from medical record review and comprised the following variables: age, sex, chest pain, systemic arterial hypertension, diabetes *mellitus*, dyslipidemia, family history of CAD, smoking, surgery type, and impaired heart valve.

Obstructive CAD was defined as luminal obstruction greater than 50% in the left main coronary artery (LCA) and obstruction greater than 70% in the other major epicardial vessels, on preoperative ICA, according to the recommendations of the Brazilian Guidelines on Valvular Heart Diseases.^1^

In our study, we dichotomized the symptoms according to the presence or absence of chest pain. Chest pain was defined as the presence of atypical or typical angina, according to the classification of the Brazilian Guidelines on Chronic Coronary Artery Disease,^14^ with two or three of the following characteristics: retrosternal discomfort or pain; triggered by exercise or emotional stress; relieved by rest or nitroglycerin use. Absence of chest pain was defined when the patient had none (asymptomatic) or only one of the above-cited characteristics (non-cardiac chest pain).

The risk factors were defined by the physicians in charge of filling out the patients' registration forms, according to their clinical judgement and the existing classifications at the time.

### Exclusion criteria

Patients with incomplete clinical data were excluded from the study.

### Statistical analysis

The categorical variables were described as frequency, being compared by use of chi-square test. The only continuous variable used in this study was age, which had a normal distribution confirmed by use of Kolmogorov-Smirnov test, was presented as mean and standard deviation and compared in the different groups by use of Student *t* test. Differences with p-value < 0.05 were considered statistically significant.

The variables associated with the outcome 'obstructive CAD' were assessed using univariate and multivariate logistic regression. The risk factors traditionally related to CAD and the variables that, on univariate analysis, showed association with obstructive CAD were included in multivariate analysis. The final model comprised the variables with statistically significant association in the multivariate model and those historically associated with CAD.

To test the calibration of the model in the derivation cohort, linear regression was used, correlating the mean estimated pretest probability (patients were divided into deciles of increasing probability of obstructive CAD) with the observed prevalence.

The predictive accuracy for obstructive CAD of the model, in both the derivation and validation cohorts, was tested by constructing the ROC curve and assessing the area under the curve.

The SPSS software (SPSS Inc., USA), version 22.0, was used for the statistical analysis.

### Score validation

The score was validated in an independent sample (validation cohort) with 294 adult patients with primary valvular heart disease, candidates for heart valve surgery from 1999 to 2005, originating from another tertiary reference hospital for heart surgery, and whose preoperative clinical and angiographic data made them eligible for the study.

## Results

From 2001 to 2014, a total of 2898 primary heart valve surgeries were recorded in adults, 1074 of whom with ICA performed in the 12 months preceding surgery were included in the study, while 362 of whom were excluded due to incomplete clinical data in the hospital registry.

The prevalence of obstructive CAD in patients with valvular heart disease and ICA in the preoperative period was 20% (145 patients).

Of the 712 patients studied, 330 (46%) were of the male sex and 382 (54%) of the female sex. Their mean age was 58 (± 12.5) years, and 145 (20%) had obstructive CAD. Chest pain was reported by 165 (23%) patients. Aortic repair surgery was performed in 291 (41%) patients, while mitral repair surgery, in 302 (42%). Double aortic-mitral repair surgery was performed in 109 (15%) patients, while combined coronary artery bypass graft surgery and valvular heart repair surgery, in 139 (20%). The prevalences of cardiovascular risk factors, impaired heart valve and obstructive CAD are shown in Table 1.

**Table 1:** Clinical characteristics of the population and according to the subgroups without and with obstructive CAD

Variables	Cohort	Without obstructive CAD	With obstructive CAD	p value
	n = 712	n = 567 (80%)	n = 145 (20%)	-
Age	58 (± 12)	55 (± 12)	66 (± 8)	< 0.001
Male sex	330 (46%)	250 (44%)	80 (56%)	0.017
Diabetes *mellitus*	96 (13%)	55 (13%)	41 (28%)	< 0.001
Arterial hypertension	493 (69%)	366 (65%)	127 (88%)	< 0.001
Dyslipidemia	338 (47%)	239 (42%)	99 (68%)	< 0.001
Family history of CAD	122 (17%)	74 (13%)	48 (33%)	< 0.001
Smoking	240 (34%)	177 (31%)	63 (43%)	0.005
Chest pain	165 (23%)	85 (15%)	80 (55%)	< 0.001
Aortic valve impairment	291 (41%)	206 (36%)	85 (59%)	< 0.001
Mitral valve impairment	302 (42%)	249 (44%)	53 (37%)	0.109
Aortic and mitral valve impairment	109 (15%)	102 (18%)	7 (5%)	< 0.001
CABG	139 (20%)	17 (3%)	122 (84%)	< 0.001

Values expressed as mean ± SD or n (%). CAD: coronary artery disease; CABG: coronary artery bypass graft. Differences with p value < 0.05 were considered statistically significant. T test was used for the variable 'age', and chi-square test, for the other variables.

Patients with obstructive CAD were older, had higher prevalence of chest pain and of traditional risk factors as compared to patients without obstructive CAD. The aortic valve, as compared to the mitral valve, was more often impaired in the former. The male sex showed a higher trend to obstructive CAD as compared to the female sex.

On univariate analysis, chest pain showed a strong association with obstructive CAD (odds ratio, 6.9; 95%CI: 4.67-10.4; p < 0.001), in addition to traditional risk factors and age. Mitral valve impairment showed no association with obstructive CAD.

The variables that associated with obstructive CAD on univariate analysis, such as traditional risk factors for atherosclerosis (age, sex, arterial hypertension, diabetes *mellitus*, dyslipidemia, family history and smoking), were entered into the multivariate analysis, in addition to aortic valve impairment, which had statistical significance. Age (p < 0.001), family history of CAD (p < 0.001) and angina (p < 0.001) were independent predictors of obstructive coronary lesion. Aortic valve impairment had no relevant association after adjusting for the other risk factors. Multivariate analysis is shown in Table 2.

**Table 2:** Univariate and multivariate analysis of risk factors for obstructive CAD

	Univariate analysis		Multivariate analysis	
Variables	Odds ratio (95%CI)	p	Odds ratio (95%CI)	p
Age	1.08 (1.06 - 1.10)	< 0.001	1.06 (1.04 - 1.09)	< 0.001
Chest pain	6.97 (4.67 - 10.41)	< 0.001	3.83 (2.44 - 6.01)	< 0.001
Family history	3.29 (2.15 - 5.03)	< 0.001	2.42 (1.46 - 3.99)	0.001
Male sex	1.56 (1.08 - 2.25)	0,17	1.29 (0.83 - 2.01)	0.255
Dyslipidemia	2.95 (2.0 - 4.35)	< 0.001	1.56 (0.99 - 2.44)	0.051
Smoking	1.69 (1.16 - 2.45)	0.006	1.34 (0.85 - 2.11)	0.198
Diabetes *mellitus*	3.67 (2.32 - 5.79)	< 0.001	1.49 (0.87 - 2.57)	0.142
Arterial hypertension	3.87 (2.29 - 6.53)	< 0.001	1.44 (0.79 - 2.62)	0.225
Aortic valve impairment	2.48 (1.71 - 2.60)	< 0.001	0.96 (0.60 - 1.53)	0.88
Mitral valve impairment	0.73 (0.50 - 1.07)	0.110	−	−

Univariate and multivariate logistic regression. Differences with p-value <0.05 were considered statistically significant.

A predictive logistic model for obstructive CAD was created based on the correlation degree between statistically significant independent predictive variables, in addition to the traditional risk factors, which, even though lacking statistical significance in the last analysis, comprised the model, because of their proven association with CAD. The logistic model is represented by the following equation:

Logit (CAD) = - 6.872 + (0.257 x male sex) + (0.066 x age) + (1.344 x chest pain) + (0.369 x hypertension) + (0.404 x diabetes) + (0.445 x dyslipidemia) + (0.297 x smoking) + (0.885 x family history of CAD)

To make clinical use easier, a score of point addition was developed, a simplification of logistic regression, where points are attributed to patients according to their clinical characteristics. One point should be added to every 5 complete yeas of life (from age zero), 1 point to each traditional risk factor (male sex, arterial hypertension, dyslipidemia, diabetes *mellitus* and smoking), 2 points to a family history of CAD, and 4 points to chest pain (Table 3).

**Table 3:** Simplified score to predict obstructive CAD

Variable	Score
Age	1 point every 5 years
Male sex	1 point
Arterial hypertension	1 point
Diabetes *mellitus*	1 point
Dyslipidemia	1 point
Smoking	1 point
Family history of CAD	2 points
Chest pain	4 points

CAD: coronary artery disease.

Patients who scored 10 points or less (estimated pretest probability < 5%) were considered to have low pretest probability, while those who scored more than 17 points (estimated pretest probability > 30%) were considered to have high pretest probability. Those who scored between 11 and 16 points comprised the intermediate group (estimated pretest probability between 5% and 30%).

The model showed an excellent correlation between estimated pretest probability and the obstructive CAD prevalence found in our population (Table 4).

**Table 4:** Prevalence of obstructive CAD according to the category of estimated pretest probability

Categories	Score	Estimated pretest probability	Observed obstructive CAD prevalence
Low probability	0-10	< 5%	2%
Intermediate probability	11-16	5 - 30%	12%
High probability	≥ 17	> 30%	49%

CAD: coronary artery disease.

**Figure 1:**
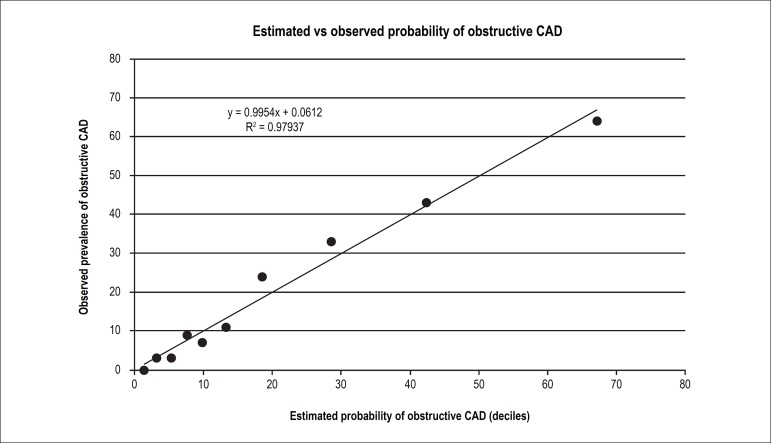
Calibration of the predictive model

To test the calibration of the predictive model, linear regression was applied correlating the estimated pretest probability (divided into deciles with increasing probability of obstructive CAD, and comprised by approximately 72 patients per decile) with the prevalence observed in the derivation cohort. A positive and significant correlation was observed between the estimated probability and the observed prevalence of obstructive CAD (R^2^ = 0.98), proving the predictive capacity of the model, represented in the 0.9954 slope of the line (close to 1.0), confirming that there is neither underestimation nor overestimation of the model tested (Figure 1).

**Figure 2:**
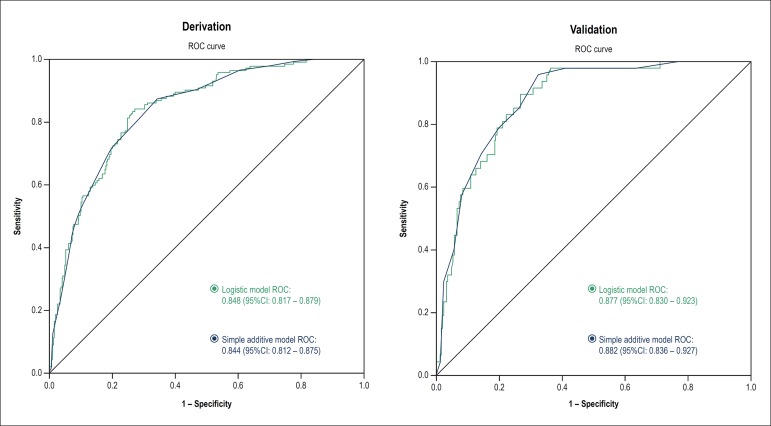
Comparison of the ROC curves of the logistic and simple additive models in the derivation and validation cohorts.

Both the logistic and the simple additive models had excellent accuracy to predict obstructive CAD in the derivation cohort, being represented by the areas under the ROC curve of 0.848 (95%CI: 0.817 - 0.879) and 0.844 (95%CI: 0.812 - 0.875), respectively (Figure 2).

To validate the models developed, we used data from a different population of 294 adult patients from another tertiary reference hospital for heart surgery, with primary valvular heart disease, candidates for heart valve surgery from 1999 to 2005. Their preoperative clinical and angiographic variables were eligible for the study.

In that validation cohort, similarly to our findings, the patients with obstructive CAD were older, mainly of the male sex and had a high prevalence of traditional risk factors. Angina occurred significantly more often in the group of patients with CAD (Table 5).

**Table 5:** Clinical characteristics of the validation cohort.

Variables	Cohort	Without CAD	With CAD	p value
	n = 294	n = 247 (84%)	n = 47 (16%)	
Age	56 (± 11)	52 (± 10)	66 (± 10)	< 0.001
Male sex	139 (47%)	106 (43%)	33 (70%)	0.002
Diabetes *mellitus*	24 (8%)	11 (4%)	13 (28%)	< 0.001
Arterial hypertension	122 (41%)	90 (36%)	32 (68%)	< 0.001
Dyslipidemia	35 (12%)	22 (9%)	13 (28%)	0.003
Family history of CAD	142 (48%)	115 (46%)	27 (57%)	0.39
Smoking	145 (49%)	116 (47%)	29 (62%)	0.18
Chest pain	125 (42,5%)	85 (35%)	39 (83%)	< 0.001
Aortic valve repair	104 (35%)	61 (59%)	43 (41%)	-
Mitral valve repair	161 (55%)	149 (93%)	12 (7%)	-
Aortic and mitral valve repair	29 (10%)	25 (86%)	4 (14%)	-

Values expressed as mean ± SD or n (%). CAD: coronary artery disease. Differences with p value < 0.05 were considered statistically significant. T test was used for the variable 'age', and chi-square test, for the other variables.

Both the logistic and simple additive models had excellent and similar accuracy to predict obstructive CAD in the validation cohort, represented by the areas under the ROC curve of 0.877 (95%CI: 0.830 - 0.923) and 0.882 (95%CI: 0.836 - 0.927), respectively (Figure 2).

## Discussion

In our cohort, the observed prevalence of obstructive CAD was 20%, lower than that of the cohorts of developed countries,^8,9^ and similar to that of the populations of developing countries.^15-19^ The prevalence of obstructive CAD in individuals aged less than 50 years was 3.3%, similar to that of other Brazilian studies. Sampaio et al. have reported a prevalence of 3.42% in a sample of 3736 patients with a mean age of 43.7 years.^12^ Kruczan et al.^11^ have shown a global prevalence of obstructive CAD of 15.9%, 6% in patients aged less than 50 years.

The patients with obstructive CAD were older, mainly of the male sex and had a high prevalence of traditional risk factors and of chest pain.

There was a univariate association between atherosclerotic risk factors, chest pain, family history, and aortic valve impairment. However, on multivariate analysis, there was no independent association between dysfunctional valve and obstructive CAD, confirming reports in the literature.^3^ Therefore, it was not entered in the logistic model. Similarly, the etiology of valvular heart disease has no independent association with CAD,^11^ but with other aggregated risk factors.

In the general population, calculators to predict and stratify CAD are widely used, and only patients with high probability and no response to clinical treatment or with tests with high-risk changes are referred for invasive stratification, while most patients with low or intermediate pretest probability being suitable for non-invasive stratification.^14^

The pretest probability of obstructive CAD is more often calculated by use of the score described in the 1970s by Diamond and Forrester,^20^ who used estimates of postmortem studies and cross-sectional studies of the North-American population. Although limited and not contemplating other cardiovascular risk factors, that score is still widely used, and continues to be recommended by the guidelines. This currently used model has been shown to overestimate the probability of CAD, and, thus, could be updated.^21,22^

For patients with valvular heart disease, there is no specific calculator to estimate obstructive CAD and, thus, to guide the preoperative period according to the calculated probability.

The AHA/ACC guideline considers CCTA a way to exclude obstructive CAD without performing ICA for patients with low or intermediate pretest probability calculated according to the criteria by Diamond and Forrester, reserving invasive stratification for patients with higher probability of CAD.^13^

In the past years, with the widespread use of CCTA for CAD stratification in the general population, several studies have tested its performance. A meta-analysis that gathered 1107 patients and 12851 coronary artery segments, has validated CCTA as a safe alternative to ICA in the preoperative period of patients with valvular heart disease.^23^ In another study assessing the preoperative period of valvular heart disease, the stratification strategy with CCTA to patients with low or intermediate pretest probability has predicted a significant cost reduction, because 28% of that study cohort would not require ICA.^4^ In addition, in 2012, an European study emphasized the importance of having a preoperative strategy, not only because it is a more comfortable diagnostic alternative for the patient, but also more inexpensive than the conventional strategy.^24^

Although ICA is gold standard for the diagnosis of obstructive lesions, it an invasive method not free from complications, such as death, vascular events (bleedings, hematomas and arterial occlusions), neurological events (ischemic and hemorrhagic) and cardiac events (arrhythmias, perforations, dissections, revascularizations, infarctions, heat failure and cardiogenic shock).^25-27^ A Brazilian study with 1916 patients has reported 190 (10.4%) complications in 175 patients.^27^ In a registry comprising 85% of the catheterization laboratories in the USA and including 1,091,557 patients, 14,736 patients (1.35%) had complications, the in-hospital mortality related to the procedure being 0.72%.^28^

To translate such data into future clinical tools, we elaborated a proposal for the preoperative assessment of patients referred for primary heart valve surgery, and applied it in the derivation cohort.

We developed a simplified easy-to-use score to stratify patients, and thus better guide the preoperative strategy. Using only clinical data, such as age, sex, chest pain and presence or absence of atherosclerotic risk factors, the pretest probability of obstructive CAD can be calculated at bedside with relative simplicity. The calculator developed in this study is available at https://connect.calcapp.net/?app=5tcj4a, and can be used in multifunctional devices.

To illustrate the use of that tool in the preoperative assessment of patients, we created arbitrarily three categories of estimated pretest probability of obstructive CAD: low, < 5%; intermediate, between 5% and 30%; and high, > 30%.

**Figure 3:**
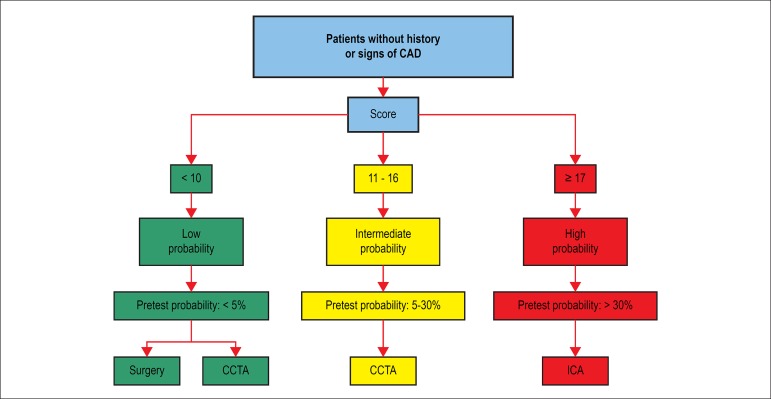
Preoperative strategy based on the use of the simple additive score and estimated pretest probability.

A patient with a score < 17 (low or intermediate probability) should be stratified conservatively, with CCTA, or even directed to heart valve surgery without additional stratification, if the probability is low, ICA being reserved for those with high pretest probability or positive CCTA for obstructive CAD (Figure 3).

In a simulation, applying the strategy proposed by the AHA/ACC guideline to our cohort, using CCTA to assess CAD in patients with low and intermediate pretest probability, we would reduce by 82% the ICA in those patients, with a total 57%reduction in the entire cohort. That strategy has a sensitivity of 99% and a specificity of 90%, using CCTA accuracy data in patients with valvular heart disease.^23^ Considering the complication rate of ICA among us,^27,28^ we would prevent 40 procedure-related complications (57% reduction).

Adopting an even more conservative strategy, with patients of low probability directed to surgery with no additional preoperative test and CCTA to assess CAD in patients of intermediate probability, we would have a 60% reduction in ICA, with sensitivity of 98% and specificity of 94%, in addition to a 61% reduction in ICA complications in our population.

That conservative strategy could result in a lack of diagnosis lower than 5% (< 2% in our cohort), which would not necessarily expose the patient to a higher risk, because cardiac catheterization itself is not free from severe complications, and it has not been clearly established that coronary artery bypass graft surgery combined with heart valve repair significantly influences patients' prognosis. In addition, ischemic complications in patients with CAD who undergo no revascularization during valve replacement are infrequent.^9,29^ Among us, the mortality of coronary artery bypass graft surgery alone ranges from 4.8% to 8.3%,^30,31^ and that rate can even triple when that surgery is combined with heart valve repair.^31^

It is worth noting that clinical predictive scores are secondary tools, and should not replace the current and previous clinical history, physical exam and previous complementary tests. Patients with a previous history of CAD, left ventricular dysfunction, evidence of myocardial ischemia on tests, or with atherosclerosis evidenced on any other exam or signs of it in other territories (such as reduced lower limb pulses, arterial stiffness and abdominal aneurysm), that increase the probability of CAD,^14^ should be treated on an individual basis.

This study had limitations. It is a retrospective analysis based on a cohort from a single tertiary center of reference, but validated in another independent cohort from another tertiary center of reference for heart surgery. Neither the previous history of CAD nor left ventricular dysfunction could be assessed, but the patients are already directed to ICA according to the recommendations of the guidelines.^1^ In addition, neither the type of valvular dysfunction (stenosis *versus* regurgitation) nor its etiology (degenerative, infectious or inflammatory) could be determined, but none of those factors was an independent predictor of CAD in a review of studies on similar populations.

## Conclusions

Obstructive CAD can be estimated based on clinical data of adult candidates for heart valve repair surgery by using a simple, accurate, calibrated, validated and easy-to-use score.

Establishing a preoperative flowchart beginning with the use of the predictive score of obstructive CAD and definition of the pretest probability group can be a more comfortable and safer strategy for the patient, preventing the indiscriminate indication of unnecessary and invasive procedures, mainly in the groups with higher probability of obstructive CAD.
